# Risk Perception of Air Pollution: A Systematic Review Focused on Particulate Matter Exposure

**DOI:** 10.3390/ijerph17176424

**Published:** 2020-09-03

**Authors:** Liliana Cori, Gabriele Donzelli, Francesca Gorini, Fabrizio Bianchi, Olivia Curzio

**Affiliations:** 1 Unit of Environmental Epidemiology and Disease Registries, Institute of Clinical Physiology, National Research Council, Via Moruzzi 1, 56124 Pisa, Italy; francesca.gorini@ifc.cnr.it (F.G.); fabrizio.bianchi@ifc.cnr.it (F.B.); olivia.curzio@ifc.cnr.it (O.C.); 2 Department of Preventive Medicine and Public Health, Food Sciences, Toxicology, and Legal Medicine, School of Pharmacy, University of Valencia, Avenida Vicente Andres Estellés s/n, Burjassot, 46100 Valencia, Spain; gabriele.donzelli@unifi.it; 3 Department of Health Science, University of Florence, 50134 Florence, Italy

**Keywords:** air pollution exposure, particulate matter (pm), environmental epidemiology, environmental pollution, perception, risk perception, public health policy, systematic review

## Abstract

The adverse health effects of exposure to air pollutants, notably to particulate matter (PM), are well-known, as well as the association with measured or estimated concentration levels. The role of perception can be relevant in exploring effects and pollution control actions. The purpose of this study was to explore studies that analyse people’s perception, together with the measurement of air pollution, in order to elucidate the relationship between them. We conducted a systematic review in accordance with the Preferred Reporting Items for Systematic Reviews and Meta-Analyses (PRISMA) guidelines. In March 2020, PubMed, EMBASE, and Scopus databases were explored in an attempt to search for studies published from 2000 to 2020. The review included 38 studies, most of which were conducted in China (*n* = 13) and the United States (*n* = 11) and published over the last four years (*n* = 26). Three studies were multicenter investigations, while five articles were based on a national-level survey. The air quality (AQ) was assessed by monitoring stations (*n* = 24) or dispersion models (*n* = 7). Many studies were population questionnaire-based, air monitoring and time-series studies, and web-based investigations. A direct association between exposure and perception emerged in 20 studies. This systematic review has shown that most of the studies establish a relationship between risk perception measurement. A broad spectrum of concepts and notions related to perception also emerged, which is undoubtedly an indicator of the wealth of available knowledge and is promising for future research.

## 1. Introduction

This systematic review is dedicated to clarifying the role of the perception of risk of air pollution, due to its relevance in the environmental and health field. The goal is to explore studies that analyse people’s perception, together with the measurement of air pollution, in order to elucidate the relationship between them. The focus is on particulate matter (PM) as its negative health effects have been demonstrated and quantified. Social, cultural, and contextual factors can influence people’s perception and the impacts on behavior, agency, self-efficacy, and the relationship with measured pollution should be clarified. This knowledge could be crucial to complementing studies on the health impacts of air pollution and improving decisions aimed at reducing human exposure. Moreover, recognizing the importance that perception holds in studies on Covid-19 [1] and its complex relationship with air pollution, this topic should be addressed and specifically investigated [2].

### 1.1. Risk Perception

Risk perception studies have been ancillary to different fields of knowledge, trying to disclose the reasons behind the behaviors of people and predict their future actions [3]. Risk perception can be defined as a person’s judgement about a risk, influenced by facts, knowledge (lay and scientific), personal preferences and attitudes (dread, trust, and interpretation of uncertainty), individual assessments (general and specific), and his/her social role (defined as “agency” by sociological disciplines; that is, the possibility/ability to act to change one’s condition). Beliefs, knowledge, values, and attitudes not only influence decisions, but also behaviors, psychophysical conditions, and exposure attitudes of people to environmental pressures [3]. Risk perception is not entirely rational since people assess risks using a mixture of cognitive skills (weighing the evidence and using reasoning and logic to reach conclusions) and emotional appraisals (intuition or imagination) [4,5,6].

In the domain of the environment and health, the study of risk perception has acquired increasing relevance as part of the knowledge required to understand social contexts and specific personal dimensions of exposure to pollutants; risk perception is fundamental in environment and health risk communication because it determines which hazards people care about and how they deal with them [7,8,9,10]. It reinforces the importance of epidemiology in evaluating the health of communities living in areas where the main perceptible sources of pollution are known, even if the evidence produced always maintains some uncertainty [11].

Complexity and uncertainty are specific features of this research activity and the inclusion of people’s perspective in the process could help to enhance the global understanding and the use of research results [9,12,13,14,15,16,17].

Nothing in risk is neutral and its perception is linked to multiple facts and motivations. Social science research in risk perception has explored and discovered how risk is understood, manufactured, or created socially, politically, and culturally [7,8,9,18,19,20,21,22,23]. Additionally, the economic evaluation of savings through which it is possible to achieve remediating high-risk areas includes risk perception calculated through the Willingness to Pay (WTP) index as an indirect indicator of health expenses [24].

### 1.2. Particulate Matter Pollution and Health

Ambient air pollution is the major environmental risk factor for the global disease burden, which has increased over the past 25 years, especially as a result of rising levels of pollution and increasing numbers of deaths from non-communicable diseases in low-income and middle-income countries [25].

Particulate matter (PM) comprises a class of pollutants consisting of a heterogeneous mixture of solid and liquid particles suspended in the air and it represents a considerable threat to health [26]. Primary PM can be released from both natural and anthropogenic sources, with road traffic considered the major source due to the erosion of pavements and abrasion of brakes and tires [27]. Secondary particles are products of the atmospheric transformation of nitrogen oxides and sulfur oxides, mainly emitted by traffic and the combustion of sulfur-containing fuels, respectively [28].

In 2013, the WHO International Agency for Research on Cancer (IARC) announced that there is sufficient evidence in humans for the carcinogenicity of outdoor air pollution and for PM in outdoor air pollution [29,30]. This understanding represented a turning point in research that focused on the health effects of inhalable PM, i.e., particles with diameters smaller than 10 (PM_10_) or 2.5 micrometers (PM_2.5_), and it was discovered that the size of particles is directly linked to their potential for causing health problems [31]. Human exposure to PM, both short and long term, has been quantitatively associated with numerous health effects, such as increased hospital admissions, emergency room visits, respiratory and cardiovascular morbidity, and mortality from cardiovascular and respiratory diseases and lung cancer [27]. Integrated exposure–response functions for each cause of death can be developed to estimate the relative risk of mortality attributable to PM. In particular, long-term exposure to PM_2.5_ was estimated to have caused 4.2 million deaths in 2015, corresponding to 7.6% of the total global mortality and making PM_2.5_ the fifth-ranking mortality risk factor [25].

For PM_10_ and PM_2.5_, concentration-response risk functions have been developed and proposed for health impact assessments. Many studies have included recommendations for actions and explorations of policy implications and the economic impact of PM reductions. Green energy policy in Europe includes air pollutant emission reduction in the motivation for reduction. Moreover, the international agreements addressing climate change consider PM in their reduction plans [32].

### 1.3. Policies

In several Asian cities, outdoor PM air pollution is increasing. In India, outdoor air pollution was responsible for more than 670,000 deaths in 2016. To deal with these health effects, several countries have launched large-scale policies, such as China [33,34] and India; in other countries like Thailand, there is a growing demand for policies to address the issue of polluted air [35]. Although in many countries there are no specific laws or protection rules, WHO guidelines are taken into consideration [28].

In China, to address the severe air pollution, the 2013 Action Plan on Prevention and Control of Air Pollution established a reduction of the PM_2.5_ concentration in Beijing, Nanjing, and Guangzhou cities by 25%, 20%, and 15%, respectively, between 2012 and 2017. After this, several plans and specific measures were implemented [36].

In the EU and US, air protection policies are similar and the reduction of air pollution is a central element. In the US, air quality (AQ) is a federal matter and the relevant policies apply to the whole country. In the EU, AQ policy is based on standards issued by the European Commission and national implementations, where Member States determine the best way to achieve them within each country. In addition, in the US, air pollution management is implemented through a combination of the AQ standard and the emission standard strategies, whereas in the EU, emission standards, emission taxation, and cost-benefit analysis are used [37].

In 2013, the European Commission adopted a Clean Air Policy Package for Europe in order to set new objectives for EU air policy and reduce the negative health impacts of air pollution, such as respiratory diseases and premature death, by almost 50% by 2030 [38].

## 2. Materials and Methods

The present systematic review was developed according to the Preferred Reporting Items for Systematic Reviews and Meta-Analyses (PRISMA) statement [39].

### 2.1. Search Strategy

This systematic review was performed by searching three different electronic databases, EMBASE, Scopus, and PubMed. Upon completing the search, a combination of Medical Subject Headings (MeSH) and non-MeSH keywords was used. Specifically, the keywords *air pollution*, *particulate matter*, *PM_10_*, *PM_2.5_*, *risk perception*, *perception*, and *health* were adopted in the following search query: (air AND pollution OR (particulate AND matter) OR PM_10_ OR PM_2.5_) AND (risk AND perception OR perception) AND health.

Only studies written in English were included and reviews, letters to the editor, and abstracts were excluded. The first search of the three databases was conducted without any time limitations. Few articles were published before 2000, so the search was limited to those published from January 2000 to March 2020.

### 2.2. Criteria for Eligibility

The following inclusion criteria were adopted:

- Perception concerns, exclusively PM exposure; if exposure refers to several pollutants or environmental matrices, PM should be specifically mentioned;
- PM levels are always measured, both directly and indirectly, and exposure refers to the specific population;
- PM measurement is only conducted outdoors;
- PM can also be included in an air quality index, AQI, while perception must always be measured, both directly and indirectly;
- Perception can be evaluated via quantitative or qualitative methods.

### 2.3. Study Selection

After removing duplicates, three researchers, who are the authors of the paper (L.C., G.D., and O.C.), independently evaluated titles and abstracts according to the eligibility criteria. The articles simultaneously selected by the three reviewers were employed in the next phase, i.e., the full text was read. The researchers thoroughly read the articles selected in the first phase in equal proportion. The reviewers individually decided to accept or reject the articles read. In cases of doubt among the three reviewers, the other two authors (F.G. and F.B.) examined the papers in order to achieve a final decision about their eligibility.

### 2.4. Data Extraction

All of the relevant data were extracted, including the author(s) name(s), publication date, title, study area, study design, age group(s) of participants, sample study, environmental exposure assessment methods, and main results. More specifically, the following information was taken into consideration: the methodology; the number and characteristics of the subjects involved in the research; how PM was measured; whether specific devices were tested; how risk perception was assessed; and which tools were used. These relevant data were included in table form to obtain a synthetic framework of all articles read in full by reviewers. This table format enabled the authors to complete a cursory overview of the materials selected in the first phase.

### 2.5. Perception Conceptual Dimensions

To be able to analyse the perception, the main methodological issue is the extent of the concept. “Perception” and “risk perception” were included in the search to be able to capture a wider variety of research in terms of methods, populations, and issues included. The perception linked to AQ measurement is intended to gather information about understanding and knowledge of the issue, psychological reactions, the capacity or availability to act as a consequence of this knowledge, and the response to public policies. Suggestions and recommendations for actions are generally presented as practical results of these studies.

To systematically examine the articles selected, the explored dimensions of perception (understanding/sensorial perception; reactions/psychological consequences; reactions/physical consequences; behaviors) were categorized by 20 features (awareness; belief; knowledge; concern; risk perception; worry; fear; outrage; familiarity; trust; annoyance; anxiety; life quality change; self-reported health symptoms; avoidance of the problem; search for information; exposure reduction; proactivity; request for action; and acceptance). This means that in each article, one or more of those features are explicitly searched as a tool for the analysis or are assumed in the concept.

## 3. Results

### 3.1. Search Results and Study Characteristics

**Figure 1:**
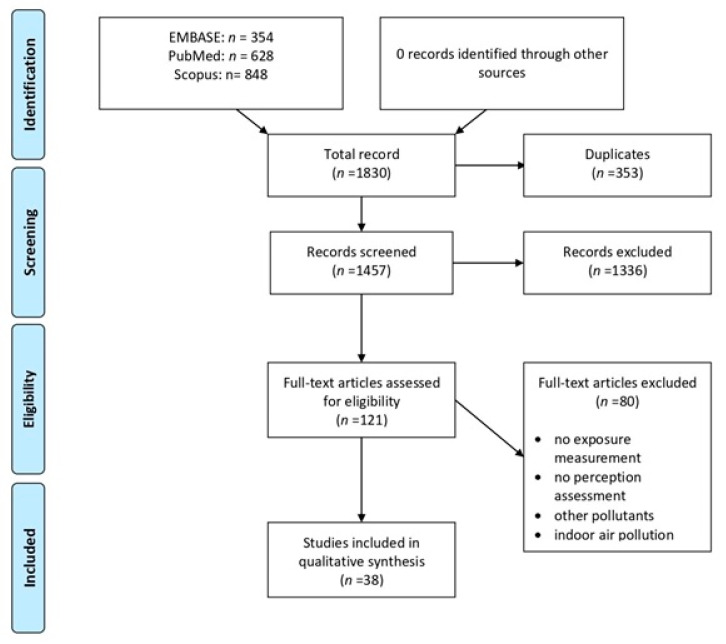
PRISMA flow diagram.

The PRISMA flow diagram in Figure 1 depicts the article selection process we followed for including studies in this review. We started by searching the three databases mentioned in the Methods section and identifying 1830 articles. From these first records, we removed 353 duplicates, leaving 1457 for further review. We reduced the number of included studies to 121 after screening the titles and abstracts and applying the following exclusion criteria:

- Studies where the main exposures referred to tobacco smoke, indoor settings, specific pollutants originating from industries, or other anthropogenic sources such as caves and mines;
- Studies evaluating health outcomes related to air pollution, but not to perception or risk perception;
- Generic studies, editorials, or abstracts in proceedings of conferences.

The remaining 121 articles underwent a full-text evaluation, bringing the total number down to 38 published articles that met our inclusion criteria. By the end of the identification process, we had removed about two-thirds of the reports from the amount we initially identified for this current study because it was impossible to know from the titles and abstracts alone whether or not they included the following criteria:

- PM exposure measurement;
- Perception assessment;
- Presence of indoor air pollution;
- Presence of other pollutants.

Table 1 summarizes the main characteristics of the studies included in this review in reverse order of publication date.

### 3.2. Geographical and Timeline Distribution

**Figure 2:**
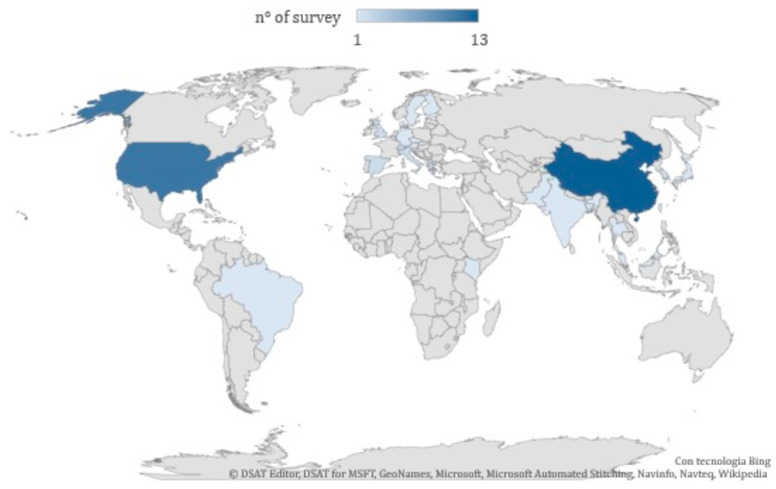
Geographical distribution of the studies included in the systematic review.

The articles included in this systematic review concern studies carried out in China (*n* = 13), the USA (*n* = 11), Greece (*n* = 3), South Korea (*n* = 2), the UK (*n* = 3), Italy (*n* = 2), Spain (*n* = 2), Switzerland (*n* = 2), Austria (*n* = 1), Bangladesh (*n* = 1), Belgium (*n* = 1), Brazil (*n* = 1), the Czech Republic (*n* = 1), Denmark (*n* = 1), Estonia (*n* = 1), Finland (*n* = 1), Germany (*n* = 1), India (*n* = 1), Japan (*n* = 1), Kenya (*n* = 1), Malaysia (*n* = 1), Pakistan (*n* = 1), Sweden (*n* = 1), Taiwan (*n* = 1) Thailand (*n* = 1), and the Netherlands (*n* = 1). Figure 2 shows the geographical distribution of surveyed countries.

Three articles are multicenter studies, two are surveys performed in seven countries [35,54], and the remaining are surveys performed in six countries [74]. Six studies were based on a national-level [41,47,49,52,60,67], three on multi-province [40,45,57], and seven on multiple-city surveys [50,51,59,63,64,71,73].

**Figure 3:**
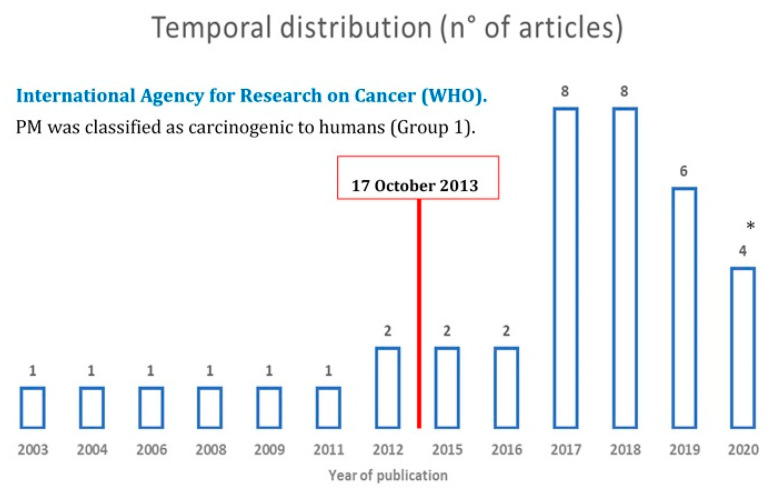
Timeline distribution of the articles included in the systematic review. * Only January, February, and March were included for the year 2020.

Looking at the timeline distribution of the articles, Figure 3 shows that research has increased over the last four years, during which time over 85% of the studies were published. As indicated in the caption for Figure 3, the year 2020 includes only the first three months of the year. The red vertical line in Figure 3 indicates the date IARC announced that PM is classified as a carcinogenic to humans (Group 1) [30].

### 3.3. Exposure Assessment of Particulate Matter (PM)

Not all studies considered the same type of PM and not all used the same methods for assessing exposure. Specifically, 18 studies only considered PM_2.5_ as an indicator of exposure to PM; 7 studies only considered PM_10_; 1 study considered PM_10_, suspended particulate matter (SPT), and total suspended particles (TSP); 2 studies considered particle number counters (PNCs); and 10 studies considered PM_2.5_ and PM_10_. Nine of the 10 studies that considered both PM_2.5_ and PM_10_ evaluated the association between pollution and survey responses using AQI, which is a six-category classification of ambient AQ for six major pollutants: carbon monoxide, ground-level ozone, nitrogen dioxide, PM_2.5_ and PM_10_, and sulfur dioxide. Regarding PM exposure assessment, the majority of studies (*n* = 24) used AQ data from official monitoring stations, while seven studies used AQ models such as the Moderate Resolution Imaging Spectroradiometer (MODIS) Aerosol Optical Depth (AOD). Only seven studies directly measured PM air concentrations (See Table 1, Column “Exposure assessment”). Two studies considered low PM level chronic exposure [52,53].

### 3.4. Study Design and Population

The 38 studies selected presented multiple designs, populations involved, and procedures. Many studies were questionnaire-based and air monitoring population studies, time series, and web-based studies.

#### 3.4.1. Population Studies

Sample sizes in general population studies ranged from 40 adult participants in community-based interviews, focus group discussions, and a community forum in Kenya [62], to 28,303 respondents, to a standardized state-based telephone survey [70], involving a total of 123,855 participants.

Six of the studies selected were not ad hoc studies, but part of major survey studies [27,40,41,48,53,66] and most of them concerned the adult population. Among the exceptions is the study of Bergstra and colleagues [51]. In this research, a cross-sectional questionnaire study was conducted for both children (2 ± 18 years) and adults (19 years and above) living in the direct vicinity of an area with heavy industry. Parents were asked to answer questions about the health of their children, with a particular focus on respiratory symptoms. Among the exceptions, a Chinese survey performed on 1000 parents 18 years old or older with children between 1 and 12 years old can also be mentioned [65].

Some research has focused on specific groups: Gany and colleagues (2016) [56] recruited taxi drivers for an air-monitoring study in 2012–2013. Another study in 2003 employed ethnographic field methods and semi-structured interviews to ask community members to identify salient risks from industrial pollution and examine whether and how perceptions differed across occupational groups (industrial workers, commercial and service sector workers, and farmers) in a Futian community [72]. A study in Malaysia included university students [46] and in a Leipzig University study, a collective of cyclists wore a unique combination of sensors that measured the particle number counts (PNC), noise, humidity, temperature, and geolocation [43].

Five studies investigated neighborhood vulnerability. In Hong Kong, a mixed-methods approach was applied to estimate neighborhood-based environmental vulnerability based on objective environmental measures and subjective environmental understanding from a local population [44]. In Wisconsin, the associations between low-level chronic exposure PM_2.5_ and cardiopulmonary health and the potential mediating or modifying effects of adverse neighborhood perceptions were examined [53]. In Nairobi, the authors made a case for participatory approaches in AQ studies, especially including those living in poor neighborhoods who may be particularly at risk from this trend [62]. In Chicago, the Community Adult Health Study studied the impact of individual, social, and built environmental factors on health and disparities in health [66]. In Seoul, Korea, 16,041 subjects aged > 20 years rated the outdoor AQ in the neighborhood [27].

#### 3.4.2. Web-Based Studies

Studies using big data from the Internet were typical of Asian countries and we selected five of them: Google trends for seven Asian megacities [35]; an Internet search engine, Baidu [47,49]; and posts analysed on Weibo (a popular microblogging system) in China [64,67].

#### 3.4.3. Procedures of the Studies

As for the procedures of the studies included in the systematic review, field studies [50,58,69,72], telephone survey studies [42,48,68,70,71,73], on-line surveys [44,54,57,61], and mixed procedure studies [53] were found. In the context of field studies, face-to-face interviews were the fundamental, but sometimes not unique, method employed to collect the data of interest to be related to the objective levels of pollution [34,42,50,55,58,59,60,66]. Considering mixed procedures and methods, we can mention Maleki’s study (2018) [53]; in this work, the protocol included in-person, audio-computer-assisted interviews; self-administered questionnaires; a physical examination; and biosample collection.

### 3.5. Risk Perception

To be able to systematically analyse the articles selected, the explored dimensions of perception were categorized by 20 features. The results of this analysis, presented in Table 2, showed that for “understanding” (44), most of the articles focused on awareness (32); for “reactions/psychological” (48), more represented risk perception (23); for “reactions/physical” (44), the declared symptoms were most represented (26); and for “behaviours” (38), exposure reduction (13) and search for information (12) were the most represented.

The details are shown in Table 2, in lines of the table including each of the features.

The studies examining understanding (44) included risk perception in 20 cases, symptoms in 23 cases, search for information in 12 cases, and exposure reduction in 13 cases.

Among the studies investigating psychological reactions (48), awareness was included in 19, symptoms in 17, search for information in 10, and exposure reduction in 12 cases.

The studies investigating physical reactions (44) also explored awareness in 24 cases, risk perception in 18 cases, search for information in 9 cases, and exposure reduction in 12 cases.

The studies analysing behaviors (38) also explored awareness in 19 cases, risk perception was considered in 16 cases, and symptoms were considered in 14 cases.

The results in terms of the association of perception, intended in the broad sense, included the 20 features examined, with measured pollution as the main outcome of this review. To examine the results in brief, see Table 3.

In only two cases, the link with air pollution was not evaluated at all: in one case [63], 14 features of perception, focusing on behaviors and socioeconomic analysis, were described and in the second case [50], the PM level measured in Hong Kong was used to build the scenarios for the questionnaire, only focusing on public awareness [50].

In five articles, measured pollution was not correlated with perception [43,48,56,71,73]. In the study by Ueberham et al. [43], cyclists were not aware of a risk posed by air pollution. In the study of Reames and Bravo [48], no association between pollution perception and air pollution exposure was observed and the findings supported the neighborhood stigma theory [66]. Physical conditions are worse in minority communities, but a disadvantaged social composition seems to independently contribute to negative perceptions of these communities. Perceptions of neighborhoods affect residents, also transcending the effects of objective conditions [72].

Gany et al. [56] carried out air monitoring in New York City taxi cabs and at roadside taxi stands, combined with a survey of taxi drivers’ knowledge related to air pollution and the associated health risks. Semenza et al. [71] measured AQ and meteorological conditions in Portland, OR and Houston, TX and 1962 subjects were interviewed by telephone about their perception and response. In Brody et al. [73], the perception of local AQ was different in Dallas and Houston and was not driven by actual readings from air monitoring stations.

An indirect influence of air pollution on perception was reported by Li et al. [40], in favor of the relation between awareness and risk perception and symptoms, and by Orru et al. [52], for which the effect of perceived exposure on symptoms and the effect of perceived exposure on disease were mediated by health risk perception.

In nine papers, AQ had a scarce influence on perception. In Huang et al. [55], the main linkage was with behavioral changes as a pre-post survey of the Youth Olympics showed that awareness and concern grew over time. In Huang et al. [59], perception was boosted by previous negative experiences with pollution. Experience was also crucial in Chen et al. [60] and Guo et al. [65], where the influence of socio-economic factors was a relevant element for explaining differences in perception. In King et al. [66], the stigma theory was utilized for explaining the linkages among awareness, risk perception, and socio-economic factors. Socioeconomic factors were also relevant in Kim et al. [27], where the declared symptoms were not explicitly correlated with perception or pollution and in Johnson et al. [68] and Rotko et al. [74], where symptoms were included and relevant. The study by Pantavou et al. (2017 and 2018) [50,58] established a direct link between symptoms and perception.

The consideration of symptoms is crucial because their linkage with perception has been widely reported in the literature. Let us consider this aspect in the following 20 articles, where a direct association between perception and measured pollution was identified, adding several further specific features.

Symptoms were a significant additional element in Misra et al. [35], where the circulation of information on the web was examined; in particular, the search for “cough” and “asthma”, together with “air pollution”, related to pollution measurement; Zakaria et al. [46] used a self-administered questionnaire in Selangor, Malaysia and Nikolopoulou et al. [69] employed a questionnaire-guide in San Diego, California to interview university students; Bergstra et al. [51] examined adults and children in an industrial area in the Netherlands; Malecki et al. [53] considered the linkage with low-level exposure in Wisconsin, USA, selecting a number of questions within a wider survey; Dons et al. [54] developed a cross-sectional questionnaire study in seven European cities; and Pantavou et al. [58] undertook a field study in Athens, associating citizen perception, behaviors, and symptoms with air pollution and dust.

In Mirabelli et al. [41], symptoms and information were investigated as a distinctive explanation because the article examined the diffusion of air pollution alerts. There were several interesting observations—in particular, that the received information declared by interviewees is a central element in perception, but it frequently does not correspond to real circulating information (i.e., 44% of citizens living in an area without AQ alerts reported being aware of the alerts). Apart from this cue, the analysis strictly correlated referred symptoms with a high perception and pollution awareness.

In Ban et al. [34], in Nanjing, China and in Wen et al. [70], in six states in the USA, together with air pollution perception, symptoms and behaviors were considered to elucidate the consequences of individual behavioral change in terms of coping with smog pollution in the first article [34] and the consequences of alerts in the second [70], Several research papers have dealt with the issue of risk communication and alerts and their effects on the population’s risk perception [41,48,69,71,73]. In Mirabelli et al. [41], knowledge of pollution and symptoms of related illnesses were more significant in areas where information about AQI was measured and disseminated. Additionally, in Reames et al. [48], pollution perception and health concerns were associated with air quality alert knowledge. The study of Wen et al. [70] showed that media alerts on air quality are a very important factor related to changes in outdoor activity. In Brody et al. [73], the authors sought to improve understanding of the major factors shaping public perceptions of air quality, concluding that perceptions appear to be influenced by the setting (urban vs. rural); state identification; and socioeconomic characteristics, such as age, race, political identification, and access to information. In the study of Semenza et al. [71], people changed behaviors when the air quality was worse, but this does not correspond with what is measured by control units.

In Dong et al. [47], AQ and perception were associated with information in the explanation because the analysis was conducted by Baidu web information dissemination and people searched for information as a reaction to the growing level of pollution.

In Tilt et al. [72], AQ and perception were associated with socioeconomic factors in the elucidation of the relationship. The study was conducted in an industrial district in China, where the people expressed concern about air and water pollution; the analysis showed the differences among industrial workers, farmers, and workers in the commercial sectors, offering an interpretation of the different points of view of the local population.

A direct association between perception and pollution has been established in a qualitative research study in a suburb in Nairobi, Kenya [62] and in a study using telephone questions and face-to-face interviews aimed at monitoring the evolution of perceived annoyance over time, related to PM in the environment [42]. Furthermore, Pu et al. [45] looked at the perceived risk and satisfaction of AQ in 31 Chinese provinces; Lu et al. [49], Wang et al. [67], and Tao et al. [64] used big data; Lu et al. (2018) [49] employed Baidu to analyse the patterns of public concern about haze and the relationship between public concern and AQ data provided by monitoring stations; Wang et al. [67] and Tao et al. [64] used Weibo to examine the content and quantity of messages referred to AQ and to construct an “Air Discussion Index”, respectively, to characterize the relationship between PM_2.5_ and social media posts; Cantuaria et al. [57] developed a cross-sectional study to assess the environmental conditions, annoyance, and health of rural residents in Denmark; and Cisneros et al. [61] studied California residents to understand their sources of AQ information, perceptions of AQ, and behaviors related to AQ.

## 4. Discussion

The main finding of the present systematic review is that 31 articles out of 38 established a relationship between perception and measured pollution, even if indirect or moderate, showing a variety of methodologies and population samples (Table 1). Regarding population samples, it must be underlined that the majority of the articles included in the systematic review did not consider the young population, when it is an integral part of the population, so this could be surveyed in the future. Given children’s vulnerability to the effects of air pollution [75], it is vital to understand how children perceive their environment. If perception surveys are performed in schools and followed by environmental and health education interventions, we could also achieve the goal of raising awareness among younger generations [13,76].

The distribution over time shows a substantial growth of publications on this topic in the last 4 years, with 26 publications between 2017 and 2020 (Figure 2); among them, the studies from China have acquired growing importance, with nine publications, while five studies were from the USA (Figure 1). This is due to an increased concern for air pollution and the implementation of control policies to be monitored and reinforced with ancillary studies. Two studies developed in China, at the national level [49], and in four Chinese megacities [64] used data collected by the USA embassy, mentioning the growing attention towards air pollution by the Chinese population that apparently the USA was boosting, offering air quality data.

As a whole, the results confirm the knowledge acquired on the relationship between air pollution and perception; in particular, that encompassed by the seminal article of Karen Bickerstaff [77], which analyzes the developments in two main disciplinary directions. First, consolidation of the socio-cultural perspective, with the contribution of anthropology, geography, and sociology, and second, a convergence between the cultural and psychological approaches. Most of the theories that maintain their validity and inform the research had already been presented [78]. Bickerstaff concluded, in particular, that the perception of risk is multi-dimensional and influenced by complex social, political, and cultural processes and that to understand how people frame the risk, it is crucial to address behavioral changes and communication. This analysis is largely valid today, with some additional elements reflected in the articles examined in this systematic review.

The diffusion of studies examining human bodies at a very sophisticated and intimate level, such as the human biomonitoring of blood, breast milk, and other specimens, or genetic tests, has led people to perceive pollution of the body and in the body in a more direct way, with relevant ethical implications [79]. The exploration of the perception of pollution in the body is a growing field of research that has developed from the experience gained and from the continuous attention to the issues of risk communication [13,80,81,82].

The choice of 20 features to summarize the different components of perception was useful for observing the frequency in the recurrences of awareness, risk perception, self-reported symptoms, and search for information, which are representative of the main conceptual areas of interest of those studies. It is also interesting to highlight that some of them were scarcely considered, such as outrage and fear, widely examined in psychological and sociological studies, but included in only one study each [34,63]; anxiety was considered in two studies [53,63] and request for actions from public authorities was considered in three cases [57,62,63].

An increasing number of studies are interpreting risk perception as a health modifier and it can be considered among the psychosocial determinants of health [83,84,85]. The term “social determinants” often evokes factors such as health-related features of neighborhoods, pointing to socioeconomic factors such as income, wealth, and education as the fundamental causes of a wide range of health outcomes [86].

Most of the studies examined in this systematic review included health considerations as self-reported symptoms, subject to personal judgement, and the linkage between perception and health symptoms was documented. This is relevant to supporting the hypothesis that risk perception plays a role as a health determinant or health effect modifier. It is interesting to note that the measured PM pollution and the short- and long-term health effects were not relevant in the studies analysed in the present review. However, we wish to emphasize that in 2013, IARC released news about the identification of PM as a carcinogenic for humans [30]. This assessment defined a step forward for scientific knowledge and the weight of air pollution within the burden of diseases became clearer, as well as its role in the distal determinants of health [87]. In the same period, there was a turning point in China in terms of the implementation of policies to protect the health of citizens from air pollution and there was a significant improvement in studies on health and air pollution [33,34].

### 4.1. Big Data Utilization and Applications

The use of search engine data and social media is changing the ways in which researchers investigate public opinion. Consequently, traditional survey research may play a less dominant role [88]. A recent review of the public’s use of big data from web searches shows that the use of Google Trends has increased dramatically in the last decade. In the process, the focus of research has shifted to forecasting changes. In contrast, in the past, the focus was merely on describing and diagnosing research trends, such as surveillance and monitoring [89]. One of the most recent applications of big data for the social sciences focused on an investigation of the phenomenon of vaccine hesitancy. Some studies have concluded that monitoring new media could be useful for detecting early signals of decreasing vaccine hesitancy and planning and targeting effective information campaigns [90,91].

This systematic review identified only five articles that studied perceived pollution using big data in the past five years. Three of them investigated perceived pollution using big data from web searches; namely, one of them used Google Trends [35] and the other two, the Baidu Index [47,49]. Although we found a correlation between the AQ level and perceived pollution in these three articles, we noticed that each study used different search terms, namely, “air pollution”, “cough”, and “asthma” [35]; “Shanghai air quality” [47]; and “haze” [49]. The other two articles collected messages from Sina Weibo, which is the most popular microblogging service in China. Both studies show that social media may be a useful proxy for measuring pollution, mainly when traditional measurement stations are unavailable, censored, or misreported [40,67].

Although these studies show promising results, several limitations should be considered, suggesting that we cannot completely abandon the research method of a questionnaire survey. In this regard, one of the main limitations is that big data can only provide an active collective public response compared to questionnaire surveys, which directly indicate public concern based on individual responses. Concerning Chinese studies, we should consider the effect of government censorship on using social media for informatics in China.

### 4.2. Communication and Recommendations

The disintermediation of communication, which has been growing exponentially in recent years, represents a relevant change in the circulation of information, with a potential relevant impact on risk perception and consequently, on health [92,93]. It is reflected in the studies examined here, with growing attention being placed on big data utilization and the continuous attention given to the utilization of results, the role of citizens, and the importance to inform and involve people to foster prevention and reduce environmental pollution.

In fact, it is clearly conceptualized by the scientific community and decision makers that the only way of managing complex problems in the environment and health domain is sharing responsibility and action to improve the quality and durability of decisions and actions. In particular, it is of utmost importance to gather information about risk perception to plan risk governance actions, including health literacy programs and communication and engagement activities, to be able to dialogue with different social actors and contribute to prevention and protection [3,21].

### 4.3. Strength, Limitations, and Future Lines of Research

To the best of our knowledge, this is the first systematic review investigating the relationship between ambient particulate matter levels and perceived pollution. In drafting this paper, we followed the PRISMA guidelines, which is one of the most appropriate tools for conducting systematic reviews. We searched three different databases to identify the highest possible number of studies and selected an extended timeframe, which allowed us to gather a significant proportion of the papers on this topic. All of the steps in the process, starting from the selection of abstracts to data extraction, were performed by three authors and not by the usual two, who have frequently consulted with each other when clarifying doubts. As for the scope of this systematic review, we are aware of the study’s limitations, mainly regarding the capability to gather all published articles on this issue. Specifically, two such limitations were the exclusion of some scientific databases and the selection of keywords. Moreover, we are aware that our decision not to consider scientific papers from gray literature may represent a limit of completeness, although, on the other hand, we believe the higher qualitative reliability of the review is reasonable and not negligible [39,94].

However, these types of limitations are inherent in all systematic reviews with similar objectives of producing new knowledge in an appropriate time period with only a few summarized articles. Regardless of the stated limitations, we believe that our work will be of interest to readers and may provide useful information for developing future lines of research focused on the investigation of social media and web search engines as predictors of air pollution peak episodes.

## 5. Conclusions

The results of this systematic review reinforce the hypothesis that AQ has an important influence on risk perception, through both indirect and direct links. There is a tendency to consider a wide spectrum of concepts and factors connected with perception, for example, behavioural changes, boosted by previous negative experiences with pollution. Experience is a key element of cognition and awareness, which can be influenced by socio-economic factors to explain differences in perception. In most articles, a direct association of perception with measured pollution was identified, with several specific features.

Unlike other types of environmental determinants, the impacts of air pollution, particularly PM, on the health of people and communities have been extensively studied, to the point of identifying a dose-response relationship. It is therefore possible to define the level of exposure of different populations and to take harm limitation measures. These measures will depend both on the actions taken by policy makers and on the willingness of people to contribute by changing personal and collective habits. From the point of view of epidemiological research, it is very interesting to understand whether and how much people are aware of the pollution that surrounds them and how it can affect their state of health. This is relevant for interpreting the existing data and for perspectives in terms of risk reduction, i.e., primary prevention.

Most of the studies examined in this systematic review include recommendations for actions and explorations of policy implications of air quality improvements. The public acceptance of mitigation policies constitutes a crucial factor for their success, necessitating ancillary actions such as health literacy programs, awareness raising campaigns, and public participation activities. The perception of people regarding exposure to air pollution and personal health is critical for evaluating the responses of communities and the acceptability of related policies. To conclude, this review may call for new studies that consider the younger segment of the population and that use big data as a source of knowledge, without renouncing the more classic methodologies of investigations. In addition, the analysis of the perception of risk as a determinant of health should be addressed. Moreover, this topic should be examined in the context of the current Covid-19 emergency and the research on the role of air pollution in the spread of the epidemic.

## Figures and Tables

**Table 1:** Characteristics of included studies.

First Author, Year	Study Area	Population Characteristics	Study Design/Objectives	Exposure Assessment	Results
P. Misra, 2020 [35]	Multi-country. Seven Asian megacities (Dhaka, Karachi, Delhi, Bangkok, Taipei, Seoul and Tokyo)	*n*/a	Perception of AQ assessed by identifying correlation of Google Trends weekly Relative Search Volume (RSV) to PM_2.5_ levels.	Moderate Resolution Imaging Spectro-radiometer (MODIS) retrieved aerosol optical depth (AOD) and angstrom exponent (AE) global satellite products.	Relationship between AQ RSV and AQ concentrations. Tendency to search for ‘air pollution’ and ‘cough’ occurs when PM_2.5_ levels are in excess and temperature is below the baseline values. RSV and remote sensing datasets could be beneficial in assessing perception and health risks during pollution episodes.
F. Li, 2020 [40]	China. 25 provinces, representative of the whole country	11,908 subjects aged 9 years or older	Questionnaire survey data. Data on individual characteristics and subjective evaluation factors (perceived environmental pollution, self-rated health, and well-being) extracted from the China Family Panel Studies for 2016.	AQI data obtained from 2401 monitoring stations in 272 prefecture-level cities in China, with data recorded hourly.	Perception of air pollution directly and negatively related to wellbeing. AQI correlated with low life satisfaction, low happiness, depression. Poor subjective health correlated with low life satisfaction, low happiness, depression.
M. Mirabelli, 2020 [41]	USA (nationwide)	12,936 men and women aged 18 and older	Cross sectional survey. Data from respondents to the 2016-2018 Consumer Styles surveys linked by geographic location and survey year to daily AQI.	County-level daily EPA’s AQ Index data. For each U.S. county with available data, it was aggregated the daily AQI data by year.	Heightened awareness of increasing pollution associated with people reporting respiratory symptoms and heart disease. Knowledge of pollution and symptoms of related illnesses more significant in areas with information and dissemination on AQI (even when the index is positive)
M. Machado, 2020 [42]	Metropolitan Region of Vitoria (MRV), Brazil	220 subjects aged 16 and older	3-year time-series survey. Relationship between perceived annoyance and PM concentration to estimate the relative risk.	Flow of monthly average sediment PM; monthly maximum and average values of PM10 and total suspended particle from eight AQ monitoring stations managed by the local environmental agency (IEMA); 3 years data (from July 2011 to July 2014).	Strong association between the perceived annoyance and different sizes of PM. An increase in air pollutant concentrations significantly associated with increasing probability of being annoyed.
M. Ueberham, 2019 [43]	City of Leipzig, Germany	66 cyclists aged 18 and older	Survey by questionnaire. Cyclists wearing sensors measuring particle counts (PNC), noise, humidity, temperature, geolocation, and subjective perception of each exposure on everyday routes for one week (*n* = 730). Smartphone application to investigate the perception of participants. Three aims to: (i) compare the multiple exposure profiles, (ii) contrast objective data and subjective individual perception, (iii) examine route decision-making and awareness of health impacts for healthier route choices.	Exposure data gathered on ordinal scale from 1 (very low) to 5 (very high). In absence of guidelines for PNC, quantiles were used to generate equally distributed classes of samples.	PNC and noise underestimated, reliable estimate for heat. The majority of cyclists underestimated their exposures to noise (84%) and PNC (80%). No significant correlation between perceived and objective exposures to noise and PNC (*p* > 0.05). In contrast, the values for heat exposure showed a significant moderate correlation (*r* = 0.68).
H. Ho, 2019 [44]	Honk Kong, China	120 adult subjects	8-item survey for rating subjective environmental statuses influencing environmental vulnerability. Estimation of neighbourhood-based environmental vulnerability based on objective and subjective environmental measures from a local population.	Map of PM_2.5_ at 500 m resolution, derived from 142 cloud-free Moderate Resolution Imaging Spectroradiometer (MODIS) Aerosol Optical Depth (AOD) datasets between 2007 and 2009.	Subjective environmental vulnerability index, SEVI, significantly associated with the local differences in mortality. Environmental conditions can directly influence people with specific illnesses and can be used to estimate community health at small-district-level.
S. Pu, 2019 [45]	China. 31 China provinces	9744 men and women aged 14 and older	Questionnaire survey data. Nationwide view on public’s air pollution risk perception and attitudes: (1) exploring spatial distribution of Chinese publics’ risk perception and attitude toward air pollution; (2) identifying characteristics of sensitive populations for air pollution; (3) analyzing which factor significantly affect publics’ attitude toward AQ.	Average annual PM_2.5_ concentration data obtained from Report on the State of the Environment in China of each province (2016).	Spatial distribution of PR, perceived risk—and SAQ, satisfaction of AQ highly concordant with actual air pollution level. PM_2.5_ has a positive direct influence on PR. PM_2.5_ has a positive moderating effect on PR and can strengthen positive correlation between PR and SAQ.
M. Zakaria, 2019 [46]	University campus, Selangor, Malaysia	180 men and women 19–22 years old	Survey by a self-administered questionnaire used to collect data on socio-demographic, AQ perception and respiratory health symptoms among university students.	Measurements of traffic-related air pollutants conducted at three sampling sites in University South Campus.	Significant associations between each level of traffic-related air pollutants and AQ perception and respiratory health symptoms (*p* < 0.05).
D. Dong, 2019 [47]	China (nationwide)	*n*/a	Web Based Time Series Study by the Baidu index to explore the relationship between actual level of air pollution and residents’ concern about air pollution. On the basis of daily data of 2068 days in Shanghai, a vector autoregression (VAR) model was built for empirical analysis.	AQ measured by the AQI reported by environmental monitoring stations. Daily AQI data to measure air pollution level in Shanghai freely available from the website of the China AQ Online Monitoring and Analysis Platform.	Local residents perceived deprivation of AQ and expressed their concern on air pollution within the day the AQ index rose. Decline of AQ in another towns, such as Beijing, raised the concern of Shanghai residents about local AQ. Rising concern in Shanghai had a beneficial impact on AQ improvement.
T. Reames, 2019 [48]	Kansas City metropolitan area, USA	2869	Space Time Data Survey to evaluate: (1) spatial distribution of PM_2.5_ and O_3_ exposures, pollution perceptions, and pollution health concerns; (2) relationships between individual- and area characteristics and PM2.5 and O3 exposure; (3) cross-sectional associations between item 2 results and pollution perception and pollution health concerns.	Census tract concentrations of PM_2.5_ and O_3_ obtained for 2009–2012 from the Community Multiscale AQ downscaler, including monitoring data obtained from the National and Local Air Monitoring Stations networks (NAMS/SLAMS).	No relationship between pollution perception and health concern, nor with air pollution exposure. Pollution perception associated with age, sex, respiratory problems, AQ alert knowledge. Health concern associated with age, sex, AQ alert knowledge, O_3_ exposure and poverty.
Y. Lu, 2018 [49]	China (nationwide)	*n*/a	Time series cross-correlation analysis. Baidu Search Index to analyze the patterns of public concern about haze from 2013 to 2017, and the dynamic relationship between public concern and AQ data.	PM_2.5_ concentration data before July 2017 from US Embassy and from 1–7 to 7-12-2017 from a data integration platform (real-time data of PM2.5 from US Embassy). Public concern investigated via web.	According to annual data, slight decrease of the weak correlation of public concern and AQ. Removing the annual trend component of time-series, the public concern resulted more sensitive to the short term fluctuation of AQ (lag), even as pollution decreased.
K. Pantavou, 2018 [50]	Athens, Greece	387 men and women aged 13 and older	Questionnaire-based study involving pedestrians at the survey sites the sites, invited to evaluate of AQ at the moment of the interview.	ATSI Dust Track 8520 aerosol monitor set for PM_10_ measurements installed in the mobile station to count PM_10_	Air dust perception related to PM_10_ concentration, affected by individual health status, smoking status, and exposure time; air pollution perception related to wind speed, gender, and health status.
A. Bergstra, 2018 [51]	Seven villages, industrial area East Vlissingen, Southwest Netherlands	2627 men and women aged 19 and older	Cross-sectional study by questionnaire to assess associations between industrial air pollution and health symptoms and risk perception about local industry.	Emission data obtained from the Emission Register.	Strong association between the perceived annoyance, some health problems and PM_2.5_ exposure. Role of parental worry about local industry in exposure-health relations for children; parents are more worried for their children than for themselves.
K. Orru, 2018 [52]	Estonia (nationwide)	1000 subjects aged 18–75	Cross-sectional study by questionnaire, with the aims to test a model that describes interrelations among air pollution, perceived pollution, health risk perception, health symptoms and diseases in 1000 Estonian residents (sample stratified by age, sex, geographical location).	Annual mean concentrations of PM_10_ in 2012 modelled with the resolution of 1 × 1 km across Estonia using a Eulerian AQ dispersion model. Data on PM_10_ at the respondent’s home address used to categorise the respondent.	No associations between level of air pollution exposure and influence perceived pollution, health risk perception, symptoms or diseases. Perceived exposure influenced health risk perception, which, in turn, influenced health symptoms and diseases, while perceived exposure affected symptoms, which influence diseases.
K. Malecki, 2018 [53]	Wisconsis, USA	2230 men and women aged 21–74	Cross-sectional study by questionnaire, to investigate associations between low-level chronic PM_2.5_ exposure and cardiopulmonary health, and the potential mediating or modifying effects of adverse neighbourhood perceptions. It used data from the Survey of the Health of Wisconsin.	Chronic PM_2.5_ exposures estimated using three-year annual average estimates derived from the United States Environmental Protection Agency’s Fused AQ Surface Downscaler model.	Association between AQ and cardiopulmonary health. Adverse perceptions of neighbourhood built environment among all participants. Significant associations between PM_2.5_ and lung function (FEV1) only among individuals with negative perceptions and increased reports of neighbourhood stressors.
E. Dons, 2018 [54]	Multi-country. Antwerp (BE), Barcelona (SP), London (UK), Orebro (SE), Rome (IT), Vienna (AU), Zurich (SW)	7622 subjects. Mean age = 40	Cross-sectional study by questionnaire to evaluate relationships between concern on health effects of air pollution and personal and environmental factors. Participants recruited to fill an online questionnaire on travel and physical activity behaviour, perceptions, attitudes on active mobility and the environment, and socio-demographic factors.	Air pollution at the residence from Europe-wide NO_2_ and PM_2.5_ maps with a 100-m resolution and available for year 2010. The NO_2_ and PM_2.5_ models explained 58.2% and 63.3% of spatial variation respectively. The European main road network was used to determine the distance of each residence to the nearest primary road.	Mean modelled air pollution and mean level of concern per city well correlated for NO_2_ (*r*^2^ = 0.75), less for PM_2.5_ (*r*^2^ = 0.49). In regression model, sex, children in the household, physical activity and NO_2_ at the home address significantly correlated to individual concern over health effects of air pollution. NO_2_ but not PM_2.5_ at the home address associated with concern over health effects of air pollution.
L. Huang, 2018 [55]	Nanjing, China	Three surveys to 250 adults each were conducted before, during and after the Youth Olympic Games (YOG) (August 16−28, 2014). Subjects aged 15 and older.	Comparative and correlation analyses of three surveys. Aim: changes in public attitudes, including their health risk perception, acceptable risk levels of air pollution, and their willingness to pay/accept for reductions in air pollution for the benefit of reducing health risks before, during, and after the Youth Olympic Games (YOG).	Respondents’ daily PM_2.5_ exposure levels calculated using an equation recommended by the US-EPA. The equation links time-activity patterns to the exposure medium concentration, normalizing by weight. The real-time ambient PM_2.5_ concentrations collected from the China Environmental Monitoring Centre (CEMC); outdoor and indoor exposure times from the questionnaire surveys.	Great differences in health perception levels, average daily PM2.5 exposure (ADD), public acceptance levels, willingness to pay and to accept compensation for air pollution before, during, and after the YOG. During the YOG, public exposed to PM_2.5_lower concentrations most accepted risks posed by haze. After YOG, residents more sensitive to haze and demanded higher AQ. A greater willingness to pay for risk reduction and to accept compensation for health-related losses.
F. Gany, 2017 [56]	New York City, USA	100 taxi drivers aged 18 and older	Survey by interview to assess taxi drivers’ knowledge, attitudes, and beliefs (KAB) about air pollution compared with direct measures of exposures. One hundred drivers completed an air pollution KAB questionnaire, and seven taxicabs participated in preliminary in-cab air sampling.	Roadside and in-vehicle levels of PM2.5 and black carbon (BC) were continuously measured over a single shift on each subject, and exposures compared with central site monitoring. Two aerosol monitors, a personal MIE DataRam 1000 and a MicroAethalometer, to measure fine PM and BC levels, respectively, inside New York City taxi cabs.	Even with general knowledge about the risks associated with occupational exposure to air pollution, taxi drivers did not consider their exposure and/or potential related risks to their health as priorities in the context of other health concerns.
M. Cantuaria, 2017 [57]	Four non-urban regions of Denmark: Anholt, Keldsnor, Lindet, Sundeved	1068 men and women aged 18 and older	Cross-sectional study to assess environmental conditions and health of residents living nearby agricultural land and animal production facilities. Residents invited via mail by answering a printed questionnaire or an online version.	Exposure assessment to 14 atmospheric pollutants. The concentrations of all pollutants, except for ammonia, were calculated using the integrated multiscale air pollution model system (THOR).	Annoyance is associated with air pollutants and noise.
K. Pantavou, 2017 [58]	Athens, Greece	1699 men and women aged 14 and older	Cross-sectional questionnaire study involving persons passing by or visiting the monitored sites, to explore AQ perception, examining their potential relationship with air pollutants concentrations, meteorological and personal variables in an urban outdoor Mediterranean environment.	Data of air pollutant concentrations were acquired from the Ministry of Environment and Energy for the air pollution stations closest to the monitoring sites	A basic relationship between AQ perception and air pollutants concentration. PM associated with dust perception; NO_x_ and CO associated with overall AQ perception. Greater perception of dusty or poor QA conditions if pollutants increased. Perception of QA influenced by age, area of residence, health symptoms and thermal sensation.
L. Huang, 2017 [59]	Beijing, Nanjingn, and Guangzhou. China	1284 men and women aged 18 and older	Cross-sectional questionnaire surveys conducted in three representative cities of China to explore how public perception and acceptable risk of air pollution can prompt individual behavioural changes and play a role in public’s response to health risks.	The respondents’ daily PM_2.5_ exposure levels, calculated by US-EPA methodology. Real-time PM_2.5_ concentrations collected from China Environmental Monitoring Centre, outdoor and indoor exposure times from the questionnaire surveys.	Risk perception different in the three cities, increases with exposure experiences. The higher measured PM level observed in women and less well-off people. Poor correlation between perception and PM_2.5_ measures.
Y. Chen, 2017 [60]	China (nationwide)	10,300	Nationwide survey. Sample selected to investigate subjective perceptions and responses to environmental pollution.	Officially weekly water quality levels at 131 monitoring sections and daily AQ levels at 109 main cities in 2008–2012 period, from the Ministry of Environmental Protection of the People’s Republic of China.	Influence by direct or indirect experience of harm and socio-economic conditions on the accuracy of individual response on 4 measured environmental parameters and attitudes. Accuracies increased low at county scale than at city scale. Accuracy on AQ higher than on water quality (47% vs 43%).
R. Cisneros, 2017 [61]	San Joaquin Valley, California, USA	744 young-adults	Survey conducted with residents to understand their sources of AQ information, perceptions of AQ, and behaviours related to AQ.	PM2.5 AQ data were downloaded from the California Air Resources Board website. The two-month PM_2.5_ mean concentrations were further grouped into three categories based on the United States NAAQS and the European AQ Standards.	Participants exposed to high PM_2.5_ concentrations perceived air pollution to be of the worst quality. AQ in the San Joaquin Valley was primarily perceived as either moderate or unhealthy for sensitive groups. Females perceived air pollution to be of worse quality compared to males.
N. Ngo, 2017 [62]	Mathare, one of the largest and oldest informal settlements in Nairobi. Kenya	40 men and women aged 24–58	Focus group discussions to understand how residents currently view air pollution. Measurements of personal exposure to PM_2.5_ by three women living in Mathare who carried pumps as they went about their daily activities.	PM_2.5_ concentrations collected on pre-weighed Teflon filters using portable air samplers carried in backpacks. Each participant pre-trained on how to use and carry the pump.	Community work on PM measurement and risk perception produced qualitative results of growing risk perception, information request on impacts, proactive protection behaviours and request for prevention measures.
J. Ban, 2017 [34]	Nanjing, China	1141 men and woman aged 16 and older	Face-to-face questionnaire survey on a random sample in Nanjing during a heavily polluted period. Objective: examine changes in individual behaviour coping with smog pollution episodes	The average PM_2.5_ concentration exceeded the national air threshold standard (75 µg/m^3^) on 52 of the 62 survey days (a rate of 83.8%), indicating frequent, serious pollution conditions in Nanjing throughout the period.	Relationship between perceived smog intensity, risk perception and behaviour change (concern behaviours), enhanced in case of negative experience or harmful health experiences (preventive actions). Higher level of awareness and risk perception for women.
C. Oltra, 2016 [63]	Four Spanish cities: Madrid, Barcelona, Zaragoza, and La Coruña	1202. Mean age = 40	Questionnaire survey, investigating how public perceptions of air pollution, risk beliefs and self reported actions limit personal exposure to air pollution across cities. Analysis to quantify association between individual variables and self-protective and information-seeking actions due to bad AQ.	Air pollution concentrations obtained from the local and regional environmental agencies.	Significant differences in subjective evaluation of local AQ, according to annoyance, physical symptoms and distress due to air pollution. Small differences in levels of self-reported attention to AQ, perceived severity and controllability beliefs. Self-reported attention to AQ levels and worry about health effects of air pollution were the most associated variables with avoid polluted streets, information seeking, change leisure activities and use face-masks.
Z. Tao, 2016 [64]	Beijing, Shanghai, Guangzhou, and Chengdu. China	513,537 messages	Survey by social network. Messages posted to Weibo (over 300 million users) to construct an “Air Discussion Index” (ADI) characterizing AQ conditions and for investigating the relationship between PM_2,5_ and social media posts.	PM_2.5_ AQ data from monitoring stations located at U.S. embassy or consulates in Beijing, Shanghai, Guangzhou, and Chengdu.	Strong correlation between the ADI and measured PM_2,5_. Social media identified as useful proxy measurement for pollution, particularly when traditional measurement stations are unavailable, censored or misreported.
Y. Guo, 2016 [65]	Whuan, China	865 men and women aged 18 or older	Survey by questionnaire for evaluating parent’s perception of AQ, by investigating the relationship between parent’s QA perception and air pollution monitoring data, and of the factors affecting parents’ perceptions.	The data of the average concentrations of primary air pollutants and the annual excellent rate of AQ in Wuhan city from the Wuhan Environmental Protection Bureau for the period 2010–2014.	General improving trend of AQ in Wuhan contrasted with the belief of deterioration in the majority of participants, indicating a significant difference between public perception and reality.
K. King, 2015 [66]	Chicago, USA	3105 men and woman aged 18 or older	Survey on a subset of the Cicago Community Adult Health Study, aiming to: (1) assess the relationship between perceived AQ and five commonly used objective measures (cancer risk, neurological risk, respiratory risk, PM_10_, O_3_; (2) investigate neighbourhood correlates of AQ perception.	Measures of ambient PM_10_ and ambient daily O_3_ (10 a.m.–6 p.m.) from the EPA’s AQ System obtained from RAND at the tract level.	Perceptions of AQ do track somewhat with objective measures of AQ, especially PM_10_ (but inversely related to O_3_ in some models). Relevant factors: socio economic status, ethnicity, gender, age and health status. Air pollution more reported by poors, blacks, hispanics, women, ills and olders.
S. Wang, 2015 [67]	China (nationwide)	435,873 messages	Analysis of big-data by mining Weibo messages about AQ role to identify AQ trends and public response in China. Comparison of the volume of AQ messages with fine particle pollution to evaluate the effectiveness of social media for complementing AQ sensors. Manual coding analysis of a sample of messages to evaluate the ability for measuring public perception, awareness, and response to pollution.	Data from the State Environmental Protection Department, which began AQ monitoring in 2012 for 74 cities.	Message volume in Sina Weibo indicative of true particle pollution levels; the messages contain rich details including perceptions, behaviors, and self-reported health effects. Social media data improve existing air pollution surveillance data, especially perception and health-related data that traditionally requires expensive surveys or interview.
M. Kim, 2012 [27]	Seoul, South Korea	16,041 men and women aged 20 or older	Cross-sectional survey study investigating (1) the relationship between measured AQ and perceived AQ in Seoul, and (2) the determinants of perceived local AQ in Seoul, focusing on individual and community-level characteristics.	Five sets of air pollution data for PM_10_, CO, SO_2_, NO_2_, O_3_ levels were obtained from 27 outdoor monitoring stations in Seoul.	Measured AQ weakly related or not related to perceived AQ. Higher risk perception associated to younger age, higher education level; no relationship with health status.
B. B. Johnson, 2012 [68]	Paterson, New Jersey. USA	441 subjects. Mean age = 50	Cross-sectional study surveyed Paterson residents to extend understanding of awareness of air pollution, sources of this awareness, and their implications for communication about air pollution.	Part of major air toxics monitoring and enforcement initiatives in Paterson, New Jersey, in 2004–2006 by the State Environmental Agency.	Perceptions of AQ not correlated with official monitoring data (U.S. AQI), but associated with health problems and social vulnerability.
M. Nikolopoulou, 2011 [69]	University of California, San Diego. USA	260 subjects. 67% aged 18–24 and 19% aged 25–34 years.	Questionnaire-guided Interviews Survey on the perception of individual exposure to different environmental stresses; microclimate, noise and PM) monitored by 260 questionnaire-guided interviews at a road construction site and a traffic site on the UC San Diego campus.	A Shinyei PPD20V particle counter sensitive to particles with a diameter larger than 1 μm was used to determine the number concentration of PM in particles per litre.	Overall, higher PM concentrations were correlated with perception of poor AQ, influenced by medical history; current smokers were the least sensitive to ambient AQ conditions.
X. Wen, 2009 [70]	Colorado, Florida, Indiana, Kansas, Massachusetts, and Wisconsin. USA	28,303 men and women aged 18 or older	Cross-sectional study, aiming at examine the association between AQ media alerts, and reductions or changes in outdoor activities among adult with asthma. Data from the 2005 Behavioral Risk Factor Surveillance System to assess reductions or changes in outdoor activities because of the perception of bad AQ, media alerts on AQI, and health professional advice to reduce outdoor activity levels.	Air pollutant concentrations collected daily by the Environmental Protection Agency and reported as AQI.	Media alerts on AQ resulted a very important factor related to changes in outdoor activity. People with lifetime asthma more than twice likely to change or reduce their outdoor activities based on their individual perception of bad AQ than people without asthma.
J. Semenza, 2008 [71]	Portland, Oregan. Houston, Texas. USA	1962. Mean age = 50.2	Survey for evaluating the effectiveness of heat and air pollution advisory systems in altering public behaviour. Subjects enrolled by random-digit-dial telephone surveys. Surveys conducted shortly after episodes of poor AQ and/or hot conditions, weekday and weekend episodes, and episodes with or without accompanying health advisories.	Temperature, relative humidity, wind speed, solar radiation, O_3_, NO, NO_2_, and PM_2.5_ data collected by the Texas Commission on Environmental Quality, the Oregon Department of Environmental Quality and a network of Portland State University monitoring stations..	Combination of behaviors when AQ is worse, but not corresponding to what is measured by the control units. No correlation between measured pollution and symptoms. Women perceive more pollution and change habits: lower-status individuals more sensitive to heat waves
B. Tilt, 2006 [72]	Futian, an industrial township in China’s southwestern province of Sichuan, China	122 workers	Survey by interview using standardised questionnaire, set up through ethnographic methods, aimed at evaluating whether and how the perceived severity of these risks varies within the community across occupational groups.	Air pollutant concentrations collected by the Chinese government, through the State Environmental Protection Administration.	Most informants perceived industrial pollution as posing considerable risk to themselves and the community. Three occupational groups (industrial, commerce and service, farmers) perceived differently in relation to socio-economic factors.
B.S.D. Brody, 2004 [73]	Two large metropolitan areas in Texas: Dallas-Fort Worth and Houston-Galveston, USA	870 adults	Survey by telephone aimed at assessing factors shaping public perception of AQ by examining spatial pattern of risk perception, role of socioeconomic characteristics, and relationship between perceived and measured air pollution. Residents surveyed as part of a state-wide telephone survey. To each respondent an average AQ based on their proximity to the nearest monitoring station was assigned.	Measured levels of AQ based on readings from continuous air monitoring stations (CAMS) in the study areas. Levels of CO, NO, O_3_, PM_2.5_ and SO_2_ recorded daily over a one-year period until August 2001.	Perceptions of local AQ different in Dallas and Huston and not driven by actual readings from air monitoring stations. Factors influencing perceptions: individual sensory experience, sense of place, proximity, neighbourhood setting, socioeconomic characteristics and media reporting.
T. Rotko, 2003 [74]	Multi-country. Six cities. Athens, Basel, Milan, Oxford, Prague and Helsinki	1736 men and women aged between 25 and 55	Analysis of a sample from EXPOLIS-European multicenter study for measurement of air pollution exposures of working age urban population during workdays. Objectives: (i) comparing levels and determinants of air pollution annoyance among the adult populations of six European cities; (ii) calculating the correlation between the perceived air pollution annoyance and the measured PM_2.5_ and NO_2_ exposures and microenvironment concentrations.	The study compares the measured pollutant concentrations (house indoor and outdoor, workplace indoor) and personal exposures to PM_2.5_ and NO_2_. PM_2.5_ was collected on two different filters: one for the working hours including commuting (personal work) and the other for the remaining hours of 48-h measurement period (personal leisure time).	No consistent or significant correlations were found between annoyance and PM_2.5_ exposure. Large variations in levels of air pollution annoyance, with the highest in traffic; significant determinants: city, self-reported symptoms, downtown residence and gender. Some associations with measured pollution emerged analysing specific correlation considering single cities, traffic, downtown living, work.

**Table 2:** Recurrence of dimensions and features related to risk perception in the selected articles.

Dimensions (Recurrence in the Selected Articles)	Features	Recurrences (*n* = 174)
Understanding/sensorial perception (*n* = 44)	Awareness = acknowledgement of the existence of a problem	32
	Belief = opinion to be exposed	5
	Knowledge = capacity to understand the existence of a problem	7
Reactions/psychological consequences (*n* = 48)	Concern = apprehension	9
	Risk perception = there is a risk for health (for me—for others)	23
	Worry = it is more that concern, linked to a depressive mood	5
	Fear = explicit reference to this emotion	1
	Outrage = main concept used by psychometric approach	4
	Familiarity = experience of the fact	5
	Trust = it is generally towards the responsible institutions	1
Reactions/physical consequences (*n* = 44)	Annoyance = feeling of negative consequences	6
	Anxiety = stress	2
	Life quality change = comfort, benefits, modification of habits	10
	Self-reported health symptoms	26
Behaviours (*n* = 38)	Avoidance of the problem	4
	Search for info	12
	Exposure reduction	13
	Proactivity = self-efficacy	4
	Request for actions = from public bodies/community	3
	Acceptance = of specific measures/policies	2

**Table 3:** Association between perception and measured air pollution. The number of articles for each kind of association is shown, with the references. Further details for each article are in Table 1.

Association	No. of Articles Showing Association	References
Association not evaluated	2	H. Ho, 2019 [44]; C. Oltra, 2016 [63]
Measured pollution not correlated with perception	5	M. Ueberham, 2019 [43]; T. Reames, 2019 [48]; F. Gany, 2017 [56]; J. Semenza, 2008 [71]; B.S.D. Brody, 2004 [73]
Indirect influence of air pollution on perception	2	F. Li, 2020 [40]; K. Orru, 2018 [52]
Scarce influence of air pollution on perception, influence by behavior, experience, socio-economic factors	9	K. Pantavou, 2018 [50]; L. Huang, 2018 [55]; K. Pantavou, 2017 [58]; L. Huang, 2017 [59]; Y. Chen, 2017 [60]; Y. Guo, 2016 [65]; K. King, 2015 [66]; B. B. Johnson, 2012 [68]; T. Rotko, 2003 [74]
A direct association between air pollution and perception is established, with a specific role of symptoms, behavior, socioeconomic factors, and information/communication	20	P. Misra, 2020 [35]; M. Mirabelli, 2020 [41]; M. Machado, 2020 [42]; S. Pu, 2019 [45]; M. Zakaria, 2019 [46]; D. Dong, 2019 [47]; Y. Lu, 2018 [49]; A. Bergstra, 2018 [51]; K. Malecki, 2018 [53]; E. Dons, 2018 [54]; M. Cantuaria, 2017 [57]; R. Cisneros, 2017 [61]; N. Ngo, 2017 [62]; J. Ban, 2017 [34]; Z. Tao, 2016 [64]; S. Wang, 2015 [67]; M. Kim, 2012 [27]; M. Nikolopoulou, 2011 [69]; X. Wen, 2009 [70]; B. Tilt, 2006 [72]

## Abbreviations

AQ	air quality
AQI	Air Quality Index
PM	particulate matter
EU	European Community
WTP	Willingness to Pay
